# Cause of fetal growth restriction during high-altitude pregnancy

**DOI:** 10.1016/j.isci.2024.109702

**Published:** 2024-04-08

**Authors:** Emily R. Brown, Dino A. Giussani

**Affiliations:** 1 Department of Physiology, Development & Neuroscience, University of Cambridge, Cambridge, UK; 2 Centre for Trophoblast Research, University of Cambridge, Cambridge, UK; 3 Cambridge Strategic Research Initiative in Reproduction; 4 Cambridge Cardiovascular Centre for Research Excellence

**Keywords:** Female reproductive endocrinology, Reproductive medicine, Women's health

## Abstract

High-altitude pregnancy increases the incidence of fetal growth restriction and reduces birth weight. This poses a significant clinical challenge as both are linked to adverse health outcomes, including raised infant mortality and the development of the metabolic syndrome in later life. While this reduction in birth weight is mostly understood to be driven by the hypobaric hypoxia of high altitude, the causative mechanism is unclear. Moreover, it is now recognized that highland ancestry confers protection against this reduction in birth weight. Here, we analyze the evidence that pregnancy at high altitude reduces birth weight and that highland ancestry confers protection, discussing mechanisms contributing to both effects.

## Highlights

• Birth weight decreases by 90.95g for every 1000m increase in altitude
• Highland ancestry decreases the birth weight reduction at altitude by half
• Fetal hypoglycaemia and hypoinsulinaemia are important in FGR at high altitude
• Uterine artery and placental metabolic adaptations protect against FGR at altitude

## Graphical abstract

**Figure:**
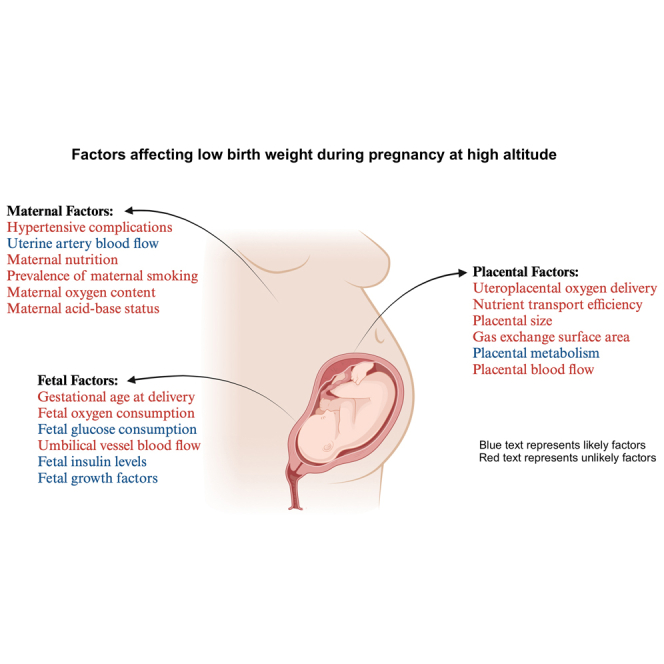

## Introduction

An estimated 81.6 million people live at high altitude (≥2,500 m) globally,^1^ and so the complications associated with high-altitude pregnancy are widespread. One of these complications is fetal growth restriction (FGR) where the fetus fails to achieve its growth potential, and its increased incidence at high altitude was first reported at least as far back as the 1950s.^2^ Despite being recognized for over 70 years, the causative mechanism mediating FGR at high altitude remains unclear. Lower birth weight has long been associated with short-term adverse health consequences and an increase in infant mortality.^3^ Further, it is now accepted that low birth weight is a surrogate marker of adverse intrauterine conditions and that it is strongly associated with a greater cardiometabolic risk in the offspring in later life.^4^^,^^5^ Therefore, understanding the mechanisms underlying FGR at high altitude will aid in its prevention and treatment, thereby contributing to a reduction in infant mortality and in cardiovascular disease for future generations of individuals living at high altitude. In this study, we have carried out a global analysis of the available literature supporting that pregnancy at high altitude reduces birth weight and that highland ancestry confers protection. Further, we have grouped the available evidence to support or refute likely underlying mechanisms contributing to both effects, including maternal, placental, and fetal factors.

## Results

### High-altitude pregnancy reduces birth weight: A global analysis

**Figure 1:**
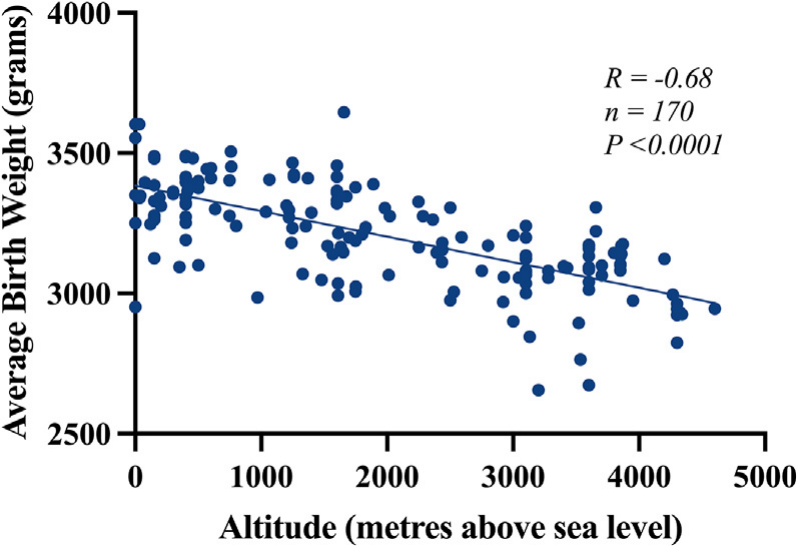
High-altitude pregnancy reduces birth weight: A global analysis Pearson product-moment correlation coefficient: R = −0.68, *n* = 170, *p* < 0.0001. Simple linear regression: Y=−0.09095X+3384. Note: If the study gave the altitude as a range, then the middle of the range was used. If the study gave only an upper or lower boundary of altitude, then the data were discounted. If the study gave birth weight adjusted for gestational age, maternal age, and parity, then this was used. If the study divided birth weights into sex or ethnicity, then the combined mean was used. This figure was created in GraphPad Prism 9.

**Figure 2:**
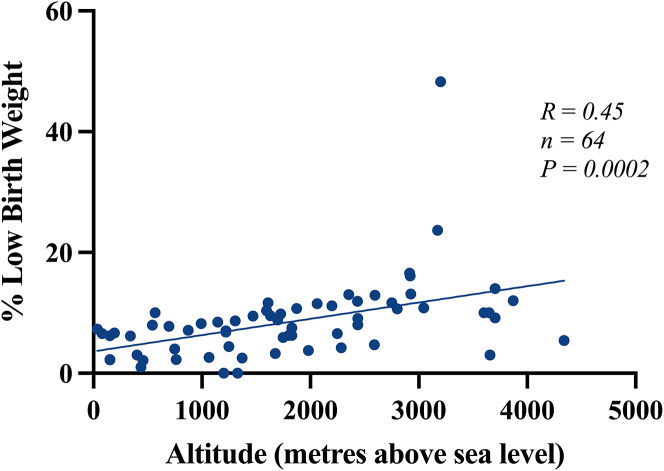
High-altitude pregnancy increases the percentage of low birth weight: A global analysis Pearson product-moment correlation coefficient: R = 0.45, *n* = 64, *p* = 0.0002. Simple linear regression: Y=0.002696X+3.631. Note: Low birth weight classified as birth weight <2,500 g. If the study gave the altitude as a range, then the middle of the range was used. If the study gave only an upper or lower boundary of altitude, then the data were discounted. The study^2^ presents an outlier with a low birth weight of 48.3% at an altitude of 3,200 m. This figure was created in GraphPad Prism 9.

The literature shows a total of 70 studies^2^^,^^6^^,^^7^^,^^8^^,^^9^^,^^10^^,^^11^^,^^12^^,^^13^^,^^14^^,^^15^^,^^16^^,^^17^^,^^18^^,^^19^^,^^20^^,^^21^^,^^22^^,^^23^^,^^24^^,^^25^^,^^26^^,^^27^^,^^28^^,^^29^^,^^30^^,^^31^^,^^32^^,^^33^^,^^34^^,^^35^^,^^36^^,^^37^^,^^38^^,^^39^^,^^40^^,^^41^^,^^42^^,^^43^^,^^44^^,^^45^^,^^46^^,^^47^^,^^48^^,^^49^^,^^50^^,^^51^^,^^52^^,^^53^^,^^54^^,^^55^^,^^56^^,^^57^^,^^58^^,^^59^^,^^60^^,^^61^^,^^62^^,^^63^^,^^64^^,^^65^^,^^66^^,^^67^^,^^68^^,^^69^^,^^70^^,^^71^^,^^72^^,^^73^^,^^74^ that support a reduction in the average birth weight with increasing altitude (Table 1). The majority focuses on populations in the United States, Bolivia, and Peru. The data show not only a marked reduction in birth weight but also an increased proportion of small-for-gestational-age (SGA) infants at altitude. A total of 66 studies^2^^,^^8^^,^^9^^,^^10^^,^^11^^,^^12^^,^^13^^,^^14^^,^^15^^,^^16^^,^^17^^,^^18^^,^^19^^,^^20^^,^^21^^,^^22^^,^^23^^,^^24^^,^^25^^,^^26^^,^^27^^,^^28^^,^^29^^,^^30^^,^^31^^,^^32^^,^^33^^,^^34^^,^^35^^,^^36^^,^^37^^,^^38^^,^^39^^,^^40^^,^^41^^,^^42^^,^^44^^,^^45^^,^^46^^,^^51^^,^^52^^,^^55^^,^^56^^,^^57^^,^^58^^,^^59^^,^^60^^,^^61^^,^^62^^,^^63^^,^^64^^,^^66^^,^^67^^,^^68^^,^^69^^,^^70^^,^^71^^,^^72^^,^^75^ reported specific birth weight values (n = 170) for any given altitude. Therefore, correlation of average values for birth weight against altitude across all multiple studies yields a significant negative relationship (Figure 1). Regression analysis reveals a significant reduction in birth weight with an increase in altitude (R = −0.68, n = 170, 95% confidence interval [CI] −0.1057 to −0.07618, *p* < 0.0001). A slope of −0.09095 indicates a 90.95 g gradual decrease in birth weight for every 1,000 m increment in altitude. Similarly, a total of 16 studies^2^^,^^7^^,^^16^^,^^18^^,^^19^^,^^21^^,^^23^^,^^27^^,^^28^^,^^29^^,^^34^^,^^43^^,^^44^^,^^45^^,^^46^^,^^75^ reported specific values (n = 64) for the prevalence of clinically defined low birth weight (<2500g) for any given altitude. Regression analysis revealed a significant increase in the prevalence of low birth weight as the altitude of pregnancy increased (R = 0.45, n = 64, 95% CI 0.001346–0.004045, *p* = 0.0002). A slope of 0.002696 indicated a 2.696% increase in the prevalence of low birth weight for every 1,000 m increment in altitude (Figure 2). This analysis further identified 12 studies^11^^,^^12^^,^^34^^,^^35^^,^^38^^,^^47^^,^^48^^,^^49^^,^^50^^,^^51^^,^^52^^,^^75^ that separated birth weight at high and low altitude according to the sex of the baby. Although male babies tend to be heavier than female babies at high- and low-altitude, analysis of the data revealed that high altitude promotes low birth weight similarly in both male (*p* < 0.0001) and female (*p* < 0.0001) babies. Therefore, high altitude does not appear to lower birth weight more in one sex compared to the other.

**Table 1:** High-altitude pregnancy reduces birth weight: A global analysis

Continent	Country/State	Reduced birth weight at high altitude	Increased low birth weight at high altitude	Increased small for gestational age at high altitude
Asia	Saudi Arabia	Al-Shehri et al.,^6^ Al-Shehri et al.,^8^ Khalid et al.,^9^ Khalid et al.,^10^ Ali et al.^11^		Al-Shehri et al.^8^
	India	Tripathy et al.^12^ Wiley et al.^13^	Wiley et al.^13^	
	Nepal		Smith et al.^75^	
	Kyrgyzstan	Reshetnikova et al.^14^		
	Turkey	Aksoy et al.^15^		
Oceania	Papua New Guinea	Primhak et al.^16^		
Africa	Rwanda	Rulisa et al.^17^		
North America	Colorado	Howard et al.^53^, Lichty et al.^2^, McCullough et al.^18^, Unger et al.^19^, Palmer et al.^20^, Jensen et al.^21^, Julian et al.^22^, Bailey et al.^23^, Zamudio et al.^24^, Schwartz et al.^25^, Tissot van Patot et al.^26^, Cogswell et al.^27^	Lichty et al.^2^, McCullough et al.^18^, Unger et al.^19^, Jensen et al.^21^, Bailey et al.^23^, Cogswell et al.^27^	Palmer et al.^20^, Julian et al.^22^
	United States	Grahn et al.^28^, Yip et al.^29^, Yip et al.^30^, Palmer et al.^31^, Zamudio et al.^32^, Zamudio et al.^33^,	Grahn et al.^28^, Yip et al.^29^	Palmer et al.^31^
South America	Argentina	Candelas et al.^54^	Candelas et al.^54^	
	Bolivia	Haas et al.^34^, Ballew et al.^35^, Kashiwazaki et al.^36^, Giussani et al.^43^, Keyes et al.^37^, Julian et al.^55^, Postigo et al.^56^, Zamudio et al.^57^, Soria et al.^58^, Bigham et al.^59^, Browne et al.^60^, Julian et al.^61^, Dávila et al.^62^, Haas et al.^47^, Julian et al.^63^, Mayhew et al.^48^, Jackson et al.^49^	Haas et al.^34^, Giussani et al.^43^	Keyes et al.^37^, Julian et al.^55^
	Peru	Sobrevilla et al.,^64^ McClung et al.,^50^ Krüger et al.,^38^ Haas et al.,^65^ Beall et al.,^39^ Haas et al.,^44^ Beall et al.,^66^ Krampl et al.,^67^ Mortola et al.,^68^ Krampl et al.,^69^ Hartinger et al.,^51^ Gonzales et al.,^45^ Gonzales et al.,^46^ Saco-Pollitt et al.,^52^ Gonzales et al.^70^	Beall et al.,^39^ Haas et al.,^44^ Gonzales et al.,^45^ Gonzales et al.^46^	
Europe	Austria	Waldhoer et al.^71^		
Worldwide	Multiple Countries	Galan et al.^72^, Galan et al.^40^, Zamudio et al.^73^, Yung et al.^41^, Grant et al.^74^, Moore et al.^42^, Zamudio et al.^7^	Grant et al.^74^, Zamudio et al.^7^	Grant et al.^74^

Studies found in the literature that show that birth weight is reduced during high-altitude pregnancy. Low birth weight was defined as birth weight <2,500 g. Small for gestational age was defined as birth weight <10^th^ percentile after adjustment for gestational age.

### Highland ancestry protection against low birth weight at altitude: A global analysis

**Figure 3:**
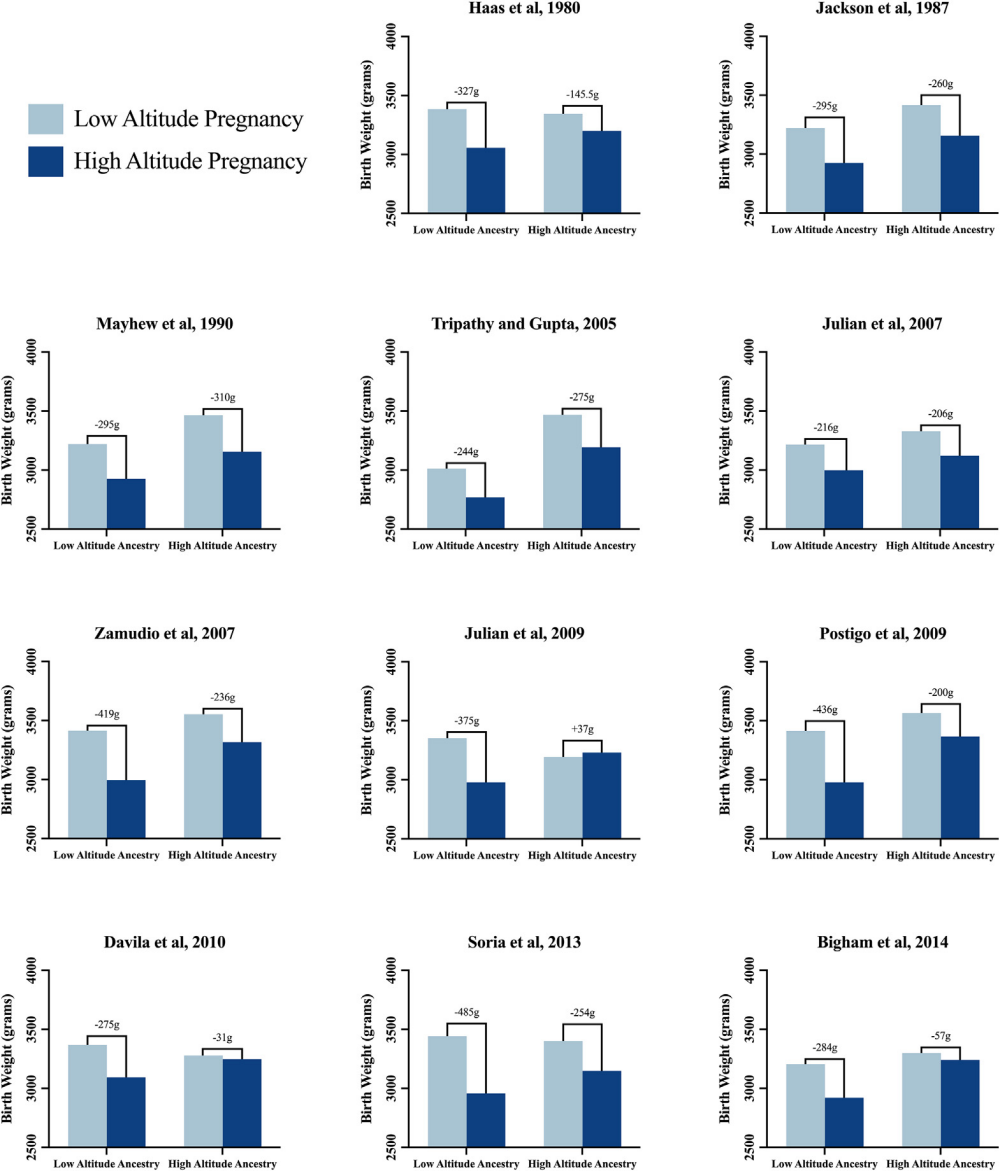
Effect of ancestry on the altitude-associated reduction in birth weight Separation of average birth weight at high and low altitude by ancestry with calculated differences reported above the bars. The decrease in birth weight from low to high altitude is less for the high-altitude ancestry group,^34^^,^^49^^,^^55^^,^^56^^,^^58^^,^^59^^,^^61^^,^^62^^,^^76^ and the birth weight at high altitude is greater for the high-altitude ancestry group than the low-altitude ancestry group.^12^^,^^48^ Birth weight even increases from low to high altitude in one high-altitude ancestry group.^61^ This figure was created in GraphPad Prism 9.

**Figure 4:**
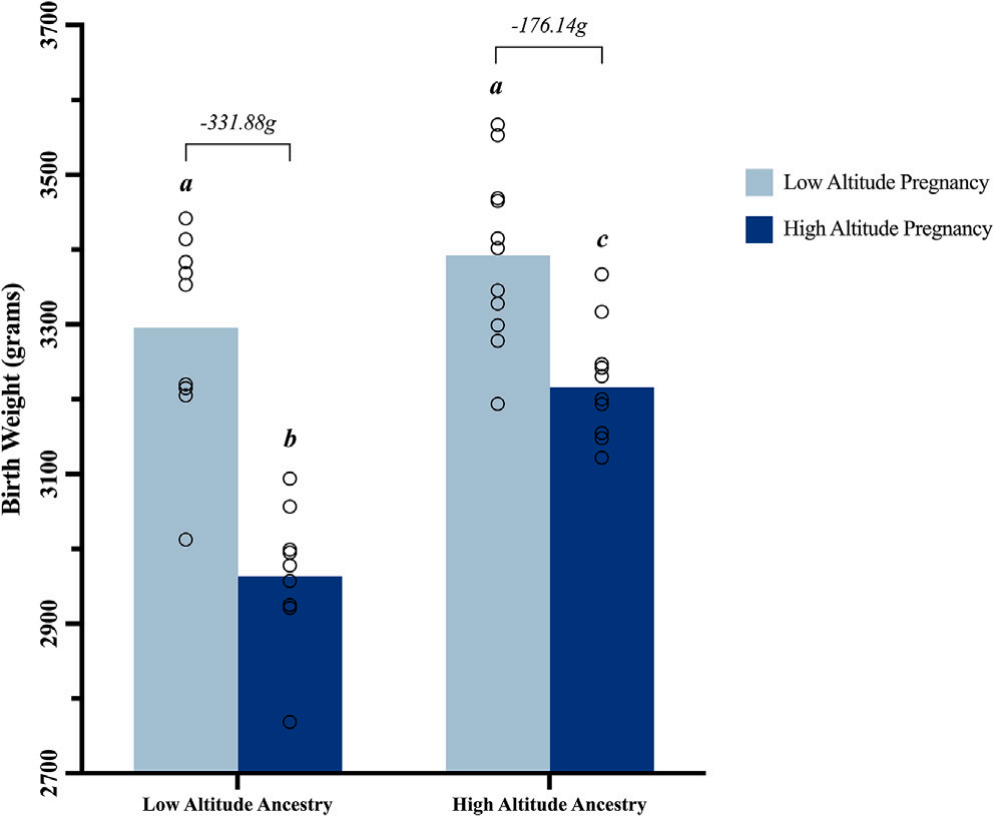
Highland ancestry protection against low birth weight at altitude: A global analysis Values are mean with overlaid dot plots for all studies comparing the effects of ancestry on the effects of high-altitude pregnancy on birth weight. On average, low-altitude ancestry populations showed a reduction of 331.88 g in birth weight during high-altitude pregnancy compared with only 176.14 g for high-altitude ancestry populations. Different letters are significantly different from each other. Two-way ANOVA showed significant effect of altitude (*p* < 0.0001) and ancestry (*p* < 0.0001) on birth weight, with a significant interaction between altitude and ancestry (*p* = 0.0176). Tukey’s multiple comparison test showed significant protection against the reduction in birth weight during pregnancy at high altitude in the high-altitude ancestry groups. This figure was created in GraphPad Prism 9.

**Figure 5:**
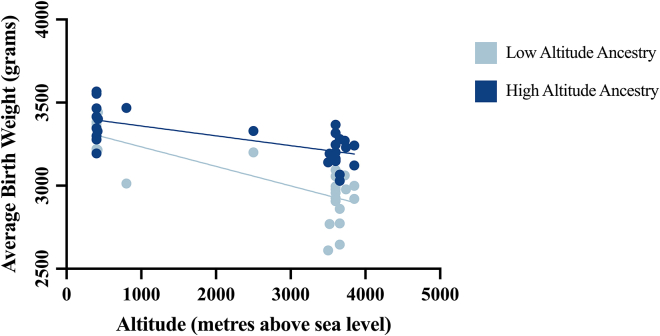
Birth weight reduction during high-altitude pregnancy separated by ancestry High-altitude ancestry Pearson product-moment correlation coefficient R = −0.6896, *n* = 30, P = <0.0001. Simple linear regression Y=−0.05911X+3419. Low-altitude ancestry Pearson product-moment correlation coefficient R = −0.8078, *n* = 30, *p* < 0.0001. Simple linear regression Y=−0.1175X+3351. The study^55^ provided birth weight measurements at three increments in altitude (416 m, 2,500 m, and 3,850 m) enabling a linear correlation. This figure was created in GraphPad Prism 9.

The literature shows 26 studies^7^^,^^12^^,^^34^^,^^42^^,^^43^^,^^49^^,^^51^^,^^55^^,^^56^^,^^57^^,^^58^^,^^59^^,^^60^^,^^61^^,^^62^^,^^75^^,^^76^^,^^77^^,^^78^^,^^79^^,^^80^^,^^81^^,^^82^^,^^83^^,^^84^^,^^85^ supporting a protective effect of highland ancestry against the altitude-associated reduction in birth weight (Table 2). Of the 26 studies, 11 included data enabling further analysis and graphical representation of the magnitude of this protection^12^^,^^34^^,^^48^^,^^49^^,^^55^^,^^56^^,^^58^^,^^59^^,^^61^^,^^62^^,^^76^ (Figure 3). On average, low-altitude ancestry populations showed a reduction of 331.88 g in birth weight during high-altitude pregnancy compared with only 176.14 g for high-altitude ancestry populations (Figure 4). Two-way ANOVA analysis reveals a significant effect of both altitude (*p* < 0.0001) and ancestry (*p* < 0.0001) on birth weight as well as a significant interaction between the two factors (*p* = 0.0176). Further analysis reveals no difference in birth weight between high- and low-altitude ancestry populations during pregnancy at low altitude (*p* = 0.1460) but a significant difference in birth weight between high- and low-altitude ancestry populations during pregnancy at high altitude (*p* < 0.0001), showing babies from mothers of highland ancestry weighing 252.7 g more than babies from mothers of lowland ancestry during pregnancy at high altitude. Assuming a linear relationship between altitude and birth weight, as in Figure 1, the low-altitude ancestry group shows a reduction in birth weight of 117.5 g compared with 59.11 g in the high-altitude ancestry group per 1,000 m increment in altitude (Figure 5). Therefore, the protection conferred by highland ancestry effectively reduces the effect of high-altitude pregnancy in lowering birth weight by almost half (49.69%).

**Table 2:** Highland ancestry protection against low birth weight at altitude: A global analysis

Continent	Country/region	Study	Main findings in support
Asia	India	Tripathy et al.^12^	At high altitude Tibetan babies had the lowest % of low birth weight compared to other ethnic groups.
		Dolma et al.^86^	Birth weight of Ladakhi and Tibetan babies at high altitude was 260 g greater than that of low-altitude ancestry babies at low altitude.
	Nepal	Smith et al.^75^	Birth weights of Sherpa babies were not different at high altitude compared to low altitude.
	Tibet	Zamudio et al.^7^	Birth weights of Tibetan babies were not different at high altitude compared to low altitude.
		Niermeyer et al.^77^	Tibetan babies weighed 294 g more than Han infants at high altitude. Two Han babies but no Tibetan babies were classified as small for gestational age at high altitude.
		Moore et al.^78^	Birth weight of Tibetan babies was 694 g more than that of Han babies at high altitude.
		Moore et al.^79^	Birth weight of Tibetan babies was 600 g more than that of Han babies at high altitude.
		Yangzom et al.^80^	Birth weight of Tibetan babies was 168 g more than that of non-Tibetan babies at high altitude. At high altitude, a lower % of Tibetan babies were classified as low birth weight or small for gestational age compared to non-Tibetan babies.
South America	Bolivia	Haas et al.^34^	Birth weight of Andean babies was 143.5 g more than that of non-Andean babies at high altitude.
		Haas et al.^81^	Birth weight of Aymaran babies was 191 g more than that of European babies at high altitude.
		Giussani et al.^43^	The altitude-associated reduction in birth weight was lower in low-income groups who tend to have greater Andean ancestry.
		Giussani et al.^82^	The altitude-associated reduction in birth weight was lower in high-altitude ancestry chicks than low-altitude ancestry chicks.
		Julian et al.^55^	Birth weight of Andean babies was 243 g more than that of European babies at high altitude. At high altitude, a lower % of Andean babies were classified small for gestational age compared to European babies.
		Vargas et al.^83^	Birth weight of Andean babies was 209 g more than that of European babies at high altitude.
		Wilson et al.^84^	Birth weight of Andean babies was 209 g more than that of European babies at high altitude.
		Zamudio et al.^76^	Birth weight of Andean babies was 322 g more than that of European babies at high altitude. The altitude-associated reduction in birth weight was 183 g greater for European babies than Andean babies.
		Bennett et al.^85^	Birth weight of Andean babies was 252 g more than that of European babies at high altitude. At high altitude, a lower % of Andean babies were classified small for gestational age than European babies.
		Julian et al.^61^	Birth weight of Andean babies was 253 g more than that of European babies at high altitude. Birth weights of Andean babies were not different at high altitude compared to low altitude.
		Postigo et al.^56^	The altitude-associated reduction in birth weight was 205 g greater for European babies than Andean babies. Birth weight of Andean babies was 389 g more than that of European babies at high altitude.
		Zamudio et al.^57^	The altitude-associated reduction in birth weight was 189 g greater for European babies than Andean babies.
		Soria et al.^58^	Birth weight of Andean babies was greatest at high altitude, followed by the birth weight of Mestizo babies and then European babies.
		Bigham et al.^59^	Birth weight of Andean babies was 321 g more than that of European babies at high altitude.
		Browne et al.^60^	Birth weights of Andean babies were not different at high altitude compared to low altitude. The % of Andean babies classified as small for gestational age was not different at high compared to low altitude.
		Dávila et al.^62^	Birth weights of Andean babies were not different at high altitude compared to low altitude. The altitude-associated reduction in birth weight was 244 g greater for European babies than Andean babies.
		Jackson et al.^49^	Birth weight of Amerindian babies was 230 g more than that of Non-Indian babies at high altitude.
	Peru	Hartinger et al.^51^	The altitude-associated reduction in birth weight was greatest in Huancayo, where people have resided for the shortest length of time, intermediate in Cuzco, and lowest in Juliaca, where people have resided for the longest length of time.
Worldwide	Multiple countries	Moore et al.^42^	Birth weight of Tibetan babies at high altitude is greater than that of Peruvian babies who in turn have greater birth weights than Coloradan babies, reflecting the length of time the populations have resided at high altitude.

Studies found in the literature that show that high-altitude ancestry protects against the altitude-associated reduction in birth weight. Low birth weight was defined as birth weight <2,500 g. Small for gestational age was defined as birth weight <10^th^ percentile after adjustment for gestational age.

### Possible causes of FGR at high altitude

**Figure 6:**
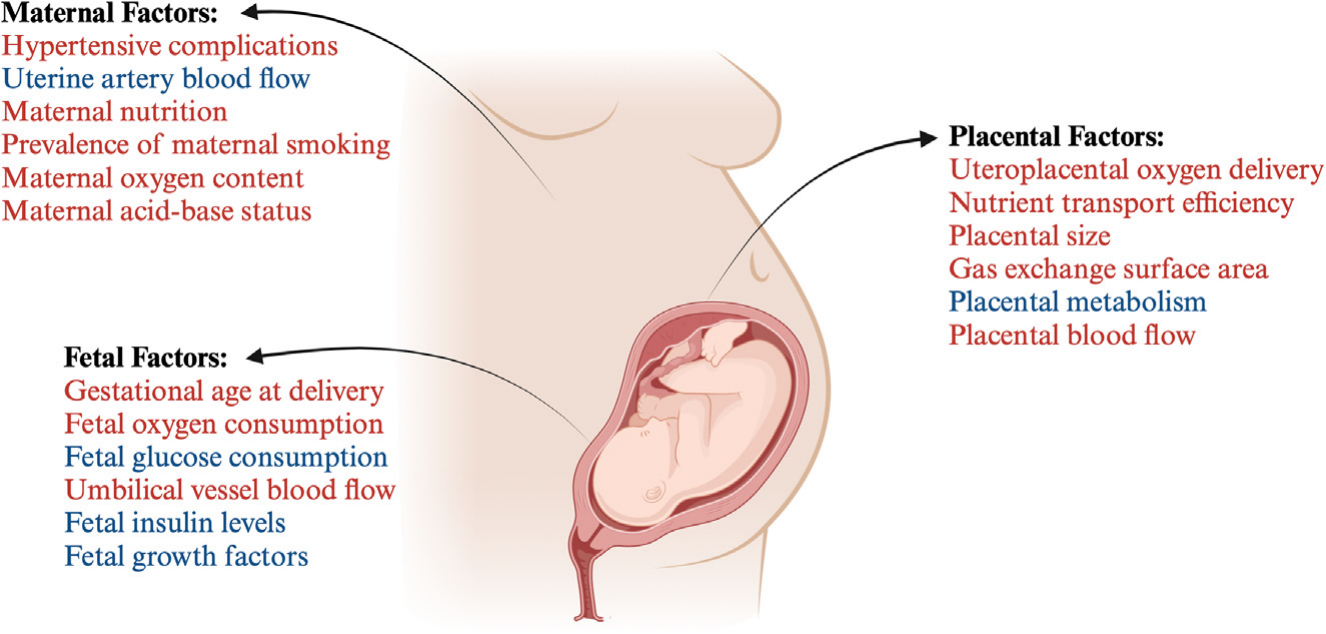
Candidate mechanisms mediating low birth weight at high altitude The weight of the evidence does not support hypertensive complications of pregnancy, differences in socioeconomic factors, prematurity, or fetal hypoxia as likely mechanisms responsible for low birth weight at high altitude. Rather, evidence in the literature suggests that reduced uterine blood flow and a switch in placental metabolism to anaerobic glycolysis maintain fetal oxygen delivery at the expense of fetal glucose delivery. Resultant fetal hypoglycemia and fetal hypoinsulinemia may promote FGR at high altitude. This may be augmented by an increase in IGFBPs at high altitude, which sequester IGFs, further decreasing the drive for fetal growth. This shift in focus away from fetal hypoxia toward placental metabolic adaptations and fetal hypoglycemia explaining low birth weight at high altitude is critical to allow research progress into identifying improved strategies for the diagnosis, prevention, and treatment of the low-birth-weight baby at high altitude. This figure was created with BioRender.com.

Over the last decade, there has been growing interest in the mechanisms contributing to the reduction in birth weight during pregnancy at high altitude, as well as those contributing to the protection of fetal growth by prolonged highland ancestry. An analysis of the literature on human studies highlights several candidate pathways. A further aim of this study was to organize potential mechanisms into maternal, placental, and fetal factors (Figure 6), providing evidence for or against any one candidate pathway (Table 3).

**Table 3:** Possible mechanisms resulting in reduced fetal growth at high altitude

**Evidence on hypertensive complications of pregnancy causing low birth weight at high altitude**			

*Evidence For:*		*Evidence Against:*	
Moore et al.^87^, Grant et al.^88^	Gestational hypertension is more common at high altitude.	Palmer et al.^20^, Jensen et al.^21^, Keyes et al.^37^	Birth weight is still lower at high altitude even when mothers with hypertensive complications are excluded.
Palmer et al.^31^	Preeclampsia is more common at high altitude.		
Zamudio et al.^76^	Mean arterial pressure is higher at high altitude.		

**Evidence on socioeconomic factors causing low birth weight at high altitude**			

*Evidence For:*		*Evidence Against:*	
		Haas et al.^34^	Maternal nutrition and smoking prevalence do not differ between low and high altitudes.
		Giussani et al.^43^	Birth weight is lower at high altitude independent of income.
		Letson et al.^89^	Low maternal weight gain and smoking account for only 34.4% of low birth weight at high altitude.

**Evidence on prematurity causing low birth weight at high altitude**			

*Evidence For:*		*Evidence Against:*	
Candelas et al.^54^	Prematurity is increased at high altitude.	Howard et al.^53^, McCullough et al.^18^, Haas et al.^34^, Moore et al.^87^, Unger et al.^19^, Zamudio et al.^24^, Grant et al.^74^	Average gestational age at delivery does not differ between low and high altitude.
		Unger et al.^19^	% Prematurity does not differ between low and high altitude.
		Jensen et al.^21^	The reduction in gestational age at delivery at high altitude is not sufficient to account for the reduction in birth weight.

**Evidence on fetal hypoxia causing low birth weight at high altitude**			

*Evidence For:*		*Evidence Against:*	
Zamudio et al.^33^	Uterine O2 delivery is 30% lower at high altitude.	Acheson et al.^90^	In sheep, fetal O2 consumption only decreases when the umbilical artery O2 saturation falls below 35% which does not occur at high altitude.
Krampl et al.^67^	Maternal PO2 is 49% lower at high altitude.	Metcalfe et al.^91^, Sobrevilla et al.^92^	Umbilical vessel PO2 is the same at low and high altitude.
Zamudio et al.^76^	Maternal respiratory alkalosis occurs at high altitude which may impair O2 unloading to the fetus.	Moore et al.^93^	Maternal O2 content at high altitude is equal or high than that at low altitude.
Browne et al.^60^	Some measures of fetal hypoxia are present even in Andeans at high altitude.	Postigo et al.^56^, Illsley et al.^94^	O2 delivery and consumption by the fetus is the same at low and high altitude.
		Zamudio et al.^57^	Uteroplacental O2 delivery is the same at low and high altitude.

**Evidence on reduced uterine artery blood flow causing low birth weight at high altitude**			

*Evidence For:*		*Evidence Against:*	
Zamudio et al.,^33^ Zamudio et al.,^76^ Julian et al.^22^	Uterine artery diameter and blood flow are reduced at high altitude.		
Dolma et al.^86^	Uterine artery diameter was significantly greater in appropriate-for-gestational-age babies compared to small-for-gestational-age babies at high altitude.		

**Evidence on alterations in placental morphology causing low birth weight at high altitude**			

*Evidence For:*		*Evidence Against:*	
Delaquerrière-Richardson et al.^95^	Increased frequency of placental infarcts in guinea pigs at high altitude.	Reshetnikova et al.^14^	Increased morphometric diffusing capacity of the placental villous membrane at high altitude.
Krüger et al.,^38^ van Patot et al.^96^	Reduced villous volume and gas exchange area at high altitude.		
Soleymanlou et al.^97^	Increased integrin-α6 expression at high altitude indicating immature trophoblast.		
Zamudio et al.^24^	Reduced GLUT1 on the placental basal membrane at high altitude.		
Yung et al.^41^	Placental endoplasmic reticulum stress and protein synthesis inhibition at high altitude.		

**Evidence on alterations in placental metabolism causing low birth weight at high altitude**			

*Evidence For:*		*Evidence Against:*	
Tissot van Patot et al.^98^	The placenta at high altitude contains higher levels of phosphocreatine and antioxidants, and lower levels of free amino acids.		
Zamudio et al.^57^	Increased anaerobic glucose consumption by the placenta at high altitude.		
Illsley et al.^94^	Oxygen delivery to the fetus remains unchanged at high altitude, whereas oxygen delivery to the placenta is reduced meaning the placenta must adapt to use less oxygen.		

**Evidence on fetal hypoglycaemia causing low birth weight at high altitude**			

*Evidence For:*		*Evidence Against:*	
Zamudio et al.^57^	Reduced glucose delivery to the fetus at high altitude.		
Illsley et al.^94^	Reduced fetal consumption of glucose at high altitude.		

**Evidence on alterations in fetal growth factors causing low birth weight at high altitude**			

*Evidence For:*		*Evidence Against:*	
Popovici et al.^99^	IGFBP-1 levels increase under conditions of hypoxia and are elevated in cases of intrauterine growth restriction.		
Krampl et al.^69^	Increased maternal IGF binding proteins at high altitude.		
Soleymanlou et al.^97^	Increased IGF binding proteins in the placenta at high altitude.		
Zamudio et al.^57^	Reduced fetal insulin levels at high altitude.		

#### Maternal factors that may lead to FGR at high altitude

Maternal factors that may lead to low birth weight during high-altitude pregnancy include hypertensive disorders, alterations in uterine blood flow, and socioeconomic factors, as well as alterations in oxygen and glucose content in the maternal circulation.

##### Hypertensive complications

Hypertensive disorders, such as preeclampsia and gestational hypertension, are known to be associated with FGR even in sea-level pregnancies.^100^^,^^101^ The hypoxia associated with highland pregnancy is suggested to affect maternal vascular adaptations to pregnancy, particularly in relation to an increase in placental vascular resistance. Upstream adverse effects of this condition result in an increase in maternal cardiac afterload and thereby mean maternal arterial blood pressure. Downstream adverse effects lead to a reduction in nutrient and oxygen delivery to the fetus, promoting FGR.^100^^,^^101^ Gestational hypertension and preeclampsia are more common at 3,100 m than 2,410 m and 1,600 m (*p* < 0.01);^87^ one study reports the incidence of preeclampsia to be 13% higher at high altitude.^31^ Additionally, maternal mean arterial pressure is significantly greater at high altitude (*p* < 0.0001)^76^ and high altitude nearly doubles the risk of gestational hypertension (OR = 1.92).^88^ Conversely, the idea that hypertensive disorders are more frequent at high altitude and cause an increase in FGR is opposed by findings that there is no difference in birth weight between infants from hypertensive or normotensive mothers during pregnancy at high altitude.^20^ Furthermore, even among normotensive women, birth weights significantly decrease at high altitude.^21^^,^^37^ It therefore seems that an increased incidence of hypertensive complications during pregnancy is not indispensable for a reduction in birth weight to occur during high-altitude pregnancy.

##### Uterine artery blood flow

High altitude may cause a reduction in uterine artery blood flow, which would limit nutrient delivery to the fetoplacental unit. In turn, the fetus would face limited growth due to nutrient and oxygen deficiency. In support, volumetric uterine artery blood flow is 1/3 lower at 3,100 m compared to 1,600 m.^33^ At high altitude, maternal plasma endothelin (ET-1) levels are elevated and nitric oxide (NOx) levels decreased, promoting a maternal constrictor phenotype, which may reduce uterine artery diameter and thereby flow.^22^ In further support, that study also showed that uterine artery blood flow was positively associated with birth weight at high altitude, and 42% of birth weight variation at high altitude could be explained by uterine artery blood flow and ET-1/NOx levels. Pregnant sheep exposed to isobaric^100^ or hypobaric^102^ gestational hypoxia also show an impaired increase in uterine blood flow and an increase in uterine vascular resistance with advancing gestation. Studies in pregnant sheep and pregnant rats also show an increase in uterine artery constrictor reactivity when gestation occurs under chronic hypoxic conditions.^102^^,^^103^ Combined, therefore, animal studies of pregnancy under the influence of iso- or hypobaric hypoxia strongly support a role for hypoxia-induced decreases in uteroplacental perfusion contributing to FGR. Uterine artery diameter and volumetric flow are lower in European compared with Andean women^76^ at high altitude, a finding that may explain the protective effect of Andean ancestry on birth weight. Consistent with this idea, Andean women had greater uterine artery diameters, cross-sectional areas, and volumetric blood flow than European women.^84^ Further, Dolma et al.^86^ have reported that Ladakhis, like Andeans, have a lower-than-expected frequency of SGA infants, particularly in families from Tibetan ancestry. Secondly, Dolma et al.^86^ found in Ladakhis that uterine artery diameter at mid-pregnancy was greater in highland women giving birth to appropriate-for-gestational-age (AGA) compared with highland women giving birth to SGA infants. Importantly, this relationship between increased uterine artery diameter and improved birth size was not present during low-altitude pregnancy. In a commentary, Moore^104^ highlights that this observation by Dolma et al.^86^ is significant because such differences in uterine artery diameter at weeks 26–28 of gestation precede any measurable effects in fetal growth. Hence, increased uterine artery diameter in prolonged high-altitude-resident populations may enable greater uterine blood flow and protect the maintenance of normal fetal growth under conditions of pregnancy at high altitude. A single-nucleotide polymorphism on the PRKAA1 gene has been discovered that relates to both infant birth weight and uterine artery diameter.^59^ The T allele was found at a higher frequency in the Andean population and linked to greater uterine artery diameter and increased birth weight. Therefore, combined, past data support that alterations in uterine blood flow likely contribute to a strong candidate mechanism underlying FGR at high altitude and the protection on birth weight conferred by high-altitude residence ancestry.

##### Socioeconomic factors

It is possible that socioeconomic factors associated with reduced birth weight, including maternal malnutrition and smoking, are more frequent at high altitude. A greater proportion of high-altitude communities do face poverty;^105^ however, maternal smoking and nutritional status are comparable at low and high altitudes,^34^ indicating that these may not be implicated in the birth weight reduction. A logistic regression analysis of 161,491 singleton births in the high-altitude state of Colorado^89^ revealed a prevalence of low maternal weight gain of 25.7% and maternal smoking of 11.6%, with a combined population-attributable fraction for low birth weight of 34.4%. Moreover, altitude independently had a population-attributable fraction for low birth weight of 12.8%.^89^ In addition, this may not reflect the true effect of high altitude as births above 4,700 feet were compared to those below, which were limited by a minimum altitude in Colorado of 3,300 feet. A separate mechanism is therefore likely to play a greater role explaining the reduction in birth weight at high altitude. In agreement, low-income, low-altitude babies have a greater mean birth weight than high-income, high-altitude babies,^43^ strongly supporting that low birth weight at high altitude occurs independent of parental economic status.

##### Maternal oxygen content and acid-base status and glycemia

The mother experiences hypobaric hypoxia at high altitude, and so maternal blood oxygen content may be lower, leading to reduced fetal oxygen delivery and growth. A 49% reduction in maternal PO_2_ at high altitude^67^ has been reported, but this is disputed with no change or even an increase in maternal O_2_ content at high altitude.^93^ It has been reported that maternal respiratory alkalosis occurs at high altitude^76^ which impairs oxygen unloading from hemoglobin. Consequently, oxygen transfer into the fetal circulation may be reduced, leading to a decrease in fetal oxygen delivery at high altitude. However, Postigo and colleagues reported preservation of human fetal oxygen delivery and consumption at high altitude,^56^ opposing this candidate mechanism. Although the underlying mechanism is unclear, studies in the Peruvian Andes^106^ and in Saudi Arabia^107^ have reported lower fasting plasma glucose in women at high altitude than in those at sea level, and in both groups the level was lower in pregnant women than in non-pregnant controls. Combined, these studies suggest that women undergoing pregnancy at high altitude have lower plasma glucose concentrations before and during pregnancy than those at sea level. Therefore, maternal hypoglycemia remains a potential maternal factor in the development of FGR during high-altitude pregnancy.

#### Placental factors that may lead to FGR at high altitude

Placental factors that may lead to low birth weight during high-altitude pregnancy include changes in uteroplacental oxygen delivery and placental nutrient transport efficiency, placental morphology, placental metabolism, and placental mitochondrial function.

##### Uteroplacental oxygen delivery and placental nutrient transport efficiency

While a 30% reduction in uterine O_2_ delivery was found at high altitude,^33^ this did not prove statistically significant. Likewise, uteroplacental O_2_ delivery was not significantly different between 400 m and 3,600 m (1.2 and 1.1 mmol.^−1^ min kg^−1^, respectively, *p* = 0.36). This was due to the increase in hematocrit at high altitude, which raised maternal arterial oxygen content to levels above those measured at low altitude.^57^ Therefore, altered uteroplacental O_2_ delivery is unlikely to play a major role in the mechanism behind the altitude-associated reduction in birth weight. Birth weight also correlates with placental system A-mediated amino acid transport capacity,^108^^,^^109^^,^^110^ and environmental hypoxia can reduce system A activity in isolated trophoblast and the mouse placenta.^111^^,^^112^ Therefore, a recent study investigated the effect of high-altitude pregnancy on human placental amino acid transport.^113^ However, there was no effect of altitude on microvillous membrane system A or L activity supporting that low birth weight in neonates of women residing at high altitude is not a consequence of reduced placental amino acid transport capacity.^113^ Conversely, there is a 40% reduction in glucose transporter 1 (GLUT1) expression at the placental basal membrane and a reduction in transferrin receptor (TfR) at high altitude.^24^ Placental basal membrane glucose transport is the rate-limiting step in glucose transfer to the fetus, and so this may result in reduced glucose delivery to the fetus. TfR is crucial for placental iron transport, and transferrin is required for fetal iron transport and efficient iron metabolism. With both placental glucose and iron transport potentially impaired at high altitude, this represents another strong candidate mechanism through which the altitude-associated reduction in birth weight may occur. A 67% reduction in TfR protein expression has also been seen in low-altitude FGR placentas compared to controls, with the lowest expression levels seen in the most severely growth restricted.^114^ This combined with the fact that maternal iron deficiency is associated with FGR^115^ indicates that iron may play a role in FGR at both low and high altitude. Conversely, the involvement of glucose transporters would appear unique to the high-altitude environment as it has been shown that placental GLUT1 expression and activity remain unchanged in FGR at low altitude.^108^^,^^116^

##### Placental morphology

An increased placental efficiency at high altitude is commonly reported, driven by either an increase in placental weight^26^^,^^38^ or maintained placental weight despite reduced birth weight.^24^^,^^57^^,^^49^ In contrast, a significant reduction in placental weight at high altitude but no change in placental coefficient has been reported,^96^ indicating less placental tissue but unchanged efficiency. Therefore, despite studies claiming placental weight as the factor that can account for the largest variation in birth weight,^76^ the effect of high altitude on placental mass is unclear and research focus has shifted to investigation of morphological and metabolic features within the placenta instead. Studies have reported that the number of cotyledons in the high-altitude placenta is half that seen at sea level,^38^ suggestive of reduced septation and villous volume. Another study reported that despite a reduction in villous volume there was no change in morphometric diffusion capacity at high altitude,^96^ supporting that gas exchange is not impaired. In fact, one study reported an increase in the morphometric diffusion capacity of the placental villous membrane at high altitude.^14^ While diffusion may not be impaired, the reduced villous volume may decrease the number of channels and transporters in the placenta and subsequently hinder the transport of non-diffusive molecules. A functional genomics study reported a 50% increase in integrin α6 expression, a marker of immature trophoblast, at high altitude.^97^ If trophoblast is immature at high altitude, placental villus formation may be impaired, which would promote a reduced placental villus volume and gas exchange area. The presence of immature trophoblast may also indicate impaired spiral artery remodeling and poor placental function at high altitude. However, integrin α6 is a hypoxia-induced gene,^117^ and so integrin α6 expression may increase due to the hypobaric hypoxia that the mother faces at high altitude rather than the increased presence of immature trophoblast. Placental morphology may also be affected by high altitude through endoplasmic reticulum (ER) stress and reduced protein synthesis. High-altitude placental tissue contained dilated ER cisternae and increased phosphorylation of p38 kinase indicating oxidative and ER stress.^41^ An observed increase in phosphorylated eIF2-α and PERK and reduction in phosphorylated AKT indicated reduced protein synthesis and translation, respectively.^41^ Therefore, changes in placental morphology at high altitude may occur due to oxidative damage-induced protein synthesis inhibition, negatively impacting fetal growth.

##### Placental metabolism and mitochondrial function

The more nutrients used by the placenta, the less available to be transferred to the fetus. Hence, changes in placental metabolism may be responsible for the altitude-associated reduction in birth weight. Studies have revealed that high-altitude placentas relative to low-altitude placentas contain greater phosphocreatine and antioxidants (taurine and inositol), but lower amino acid levels.^98^ This indicates that high-altitude placentas adapt to chronic hypoxia by altering their metabolism to store energy as phosphocreatine and to enhance their capacity to resist oxidative stress. Another indication that placental metabolism is altered at high altitude is the preferential anaerobic glucose consumption by the placenta that preserves oxygen for the fetus.^57^ This is likely driven by the activation of pyruvate dehydrogenase 1 by HIF-1, which inhibits the mitochondrial citric acid cycle and reduces mitochondrial oxygen consumption.^118^ Maternal arterial-to-venous plasma glucose concentration is significantly greater at high altitude,^57^ suggesting that placental glucose utilization increases at high altitude to meet the placental ATP requirement in the face of less efficient anaerobic glycolysis. It is possible that, in doing so, the placenta promotes fetal hypoglycemia, which may be exacerbated by underlying maternal hypoglycemia in the highland mother, which may in turn decrease fetal growth. Metabolic adaptation of the placenta at high altitude is supported by the discovery that metabolic changes are apparent in individuals protected by their high-altitude ancestry against the altitude-associated reduction in birth weight. The TT genotype for the rs1345778 single-nucleotide polymorphism in the α1 catalytic subunit of adenosine monophosphate-activated protein kinase (AMPK) is associated with an increased birth weight at high altitude, and this T allele is found at a higher rate in Andean (88%) compared with European women (73%).^59^ AMPK is involved in regulating the mTOR pathway, and in individuals with the protective TT genotype, 12 mTOR genes are differentially transcribed. The mTOR pathway is central to placental metabolic control and has been implicated in cases of FGR unrelated to altitude. Other studies have reported that placentas of Tibetan women living at high altitude are protected from labor-induced oxidative stress compared to those of other highland residents.^119^ Sferruzzi-Perri and colleagues^120^ have shown that, in the mouse placenta, mitochondria adapt their use of oxygen and nutrients to best support both placental growth and function, as well as fetal development, during normal and hypoxic conditions. Further, Liu et al.^121^ discovered that human placental mitochondrial respiration was greater in high-altitude than in lower-altitude Tibetan women. The authors suggested that the increased mitochondrial respiration in placentas from Tibetan women with a prolonged high-altitude residence ancestry may represent an adaptive response to remedy the increased energy demand and oxidative stress imposed by labor in an oxygen-deprived environment. Consequently, it is very likely that alterations in placental metabolism and placental mitochondrial function play a central role in both the reduction in birth weight at altitude and the protection against low birth weight conferred by highland ancestry.

#### Fetal factors that may lead to FGR at high altitude

Fetal factors that may lead to low birth weight during high-altitude pregnancy include changes in fetal oxygen, fetal glucose, fetal insulin levels, and premature birth.

##### Fetal hypoxia, fetal glucose, and fetal insulin levels

As the mother faces hypobaric hypoxia at high altitude, it may seem obvious to assume that the fetus also faces hypoxia. However, alterations in placental metabolism maintain adequate oxygen delivery to the fetus, a view reinforced by the findings that umbilical pO2,^91^ fetal scalp capillary blood oxygen tension,^92^ and human fetal oxygen delivery and consumption are all preserved at high altitude.^56^ Opposing findings come from reports of fetal hypoxia markers at high altitude, including lower fetal heart rate and cerebral artery pulsatility index.^60^ Conversely, a 35% reduction in fetal glucose consumption at high altitude has been reported.^94^ Further, studies confirm reduced glucose delivery and consumption by the fetus at high altitude (*p* < 0.0001) due to reduced umbilical glucose concentration.^57^ Therefore, the fetus at high altitude is hypoglycemic, and reduced glucose rather than oxygen availability is more likely to promote FGR and account for the reduction in birth weight during highland pregnancy. Further, umbilical vein diameter and umbilical blood flow are reduced at high altitude,^56^ and umbilical blood flow is lower in European than Andean pregnancies.^56^ This reduction in umbilical blood flow will limit delivery of substrates to the fetus which in turn will reduce fetal growth. Reduced umbilical vessel blood flow may therefore compound the decrease in glucose delivery to the fetus at high altitude. In turn, insulin is released in response to glucose, and so the hypoglycemia that the fetus faces at high altitude may result in reduced fetal insulin levels.^57^ Insulin acts to drive fetal growth, which raises the possibility that fetal hypoinsulinemia may underlie a mechanism promoting FGR at high altitude. Like insulin, insulin-like growth factors (IGFs) also drive fetal growth. IGF binding proteins (IGFBPs) bind to IGFs in the blood stream and prevent them from binding to their receptors. The majority of IGFBPs inhibit the actions of IGFs^122^ and so result in reduced growth. Fetal hepatocytes cultured under hypoxic conditions undergo a 2.5-fold and 3-fold increase in IGFBP-1 and IGFBP-3 levels, respectively,^99^ leading to suggestions that this is the molecular cause for FGR resulting from placental insufficiency. However, the levels of hypoxia used in that study were far below physiological levels at 2% O_2_ and no change in IGFBP-1 was observed when raised to 10% O_2_. While the fetus at high altitude does not face hypoxia, IGFBP-1 is normally inhibited by insulin,^123^ and so the hypoinsulinemia that the fetus faces at high altitude may allow the IGFBP-1 level to rise and induce FGR in a similar manner. Although fetal IGFBP levels have not been measured directly, there is an increase in maternal IGFBP-1 at high altitude.^69^ Interestingly, this increase became significant from week 25 of gestation which closely mirrors the onset of reduced fetal growth in the third trimester. In addition to IGFBP-1 increasing in expression at high altitude, there is a 2.5-fold increase in IGFBP-3 in the placenta at high altitude.^97^ As human studies have only measured maternal and placental IGFBP levels, further studies into human fetal IGFBP levels are necessary to confirm whether this also occurs in the fetal compartment.

##### Premature birth

It is possible that the stress of the hypobaric hypoxia that mothers face at high altitude may induce preterm delivery, and so a reduction in gestational age at birth may be responsible for the reduction in birth weight. While an increase in prematurity at high altitude has been reported,^54^ the vast majority of studies refute this.^18^^,^^19^^,^^24^^,^^34^^,^^53^^,^^57^^,^^74^^,^^87^ In one study that claimed an increase in the incidence of prematurity at high altitude, birth weight was used as an index of prematurity, and so it is more likely that they simply observed smaller babies at high altitude rather than an increased rate of prematurity.^124^ Therefore, gestational age at delivery is not significantly decreased at high altitude and cannot account for the altitude-associated reduction in birth weight.

## Discussion

This global evaluation of the available literature used regression analysis to calculate a 90.95 g decrease in birth weight and a 2.69% increase in incidence of low (<2,500 g) birth weight for every 1,000 m increment in altitude in studies across the world. We found no evidence to support any differential effect of high altitude in reducing birth weight in male and female babies. Furthermore, this global analysis compiled evidence to support that families of highland ancestry have significant protection against the high-altitude-induced FGR, showing only a 59.11 g reduction in birth weight in the high-altitude ancestry group compared with a 117.5 g reduction in birth weight in the low-altitude ancestry group per 1,000 m increment in altitude. This effectively equates to a 50% protection against the effect of high altitude in reducing birth weight in families of high- compared to low-land ancestry.

The weight of the evidence does not support hypertensive complications of pregnancy, differences in socioeconomic factors, prematurity, or fetal hypoxia as likely mechanisms responsible for low birth weight at high altitude. Rather, evidence in the literature suggests that reduced uterine blood flow and a switch in placental metabolism to anaerobic glycolysis that maintains fetal oxygen delivery at the expense of fetal glucose delivery may be more likely mechanisms. This effect may be compounded by the woman being hypoglycemic herself during pregnancy at high altitude. Resultant fetal hypoglycemia and fetal hypoinsulinemia may promote FGR at high altitude. This may be augmented by an increase in IGFBPs at high altitude, which sequester IGFs, further decreasing the drive for fetal growth. Since families of highland ancestry also show beneficial adaptations in uterine artery blood flow and in placental metabolism designed to protect fetal growth, this further reinforces these pathways relative to others as the most important candidate mechanisms in mediating low birth weight at high altitude.

This shift in focus away from fetal hypoxia toward placental metabolic adaptations and fetal hypoglycaemia explaining low birth weight at high altitude is critical to allow research to progress into identifying improved strategies for the diagnosis, prevention, and treatment of the low-birth-weight baby at high altitude.

### Limitations of the study

This was a retrospective global analysis of the available data in the literature. Therefore, the work is limited by the availability of the data in a format that was usable for such analysis. This is particularly relevant to the analysis of the protection conferred by high-altitude ancestry, where data are less available and the application of a linear relationship between altitude and birth weight was assumed for low- and high-altitude ancestry groups. Furthermore, available datasets were often given in different formats. Where the altitude was given as a range, the mid-point was used. Where an upper or lower boundary was given, the data were discounted due to being unable to predict the spread of data. While birth weight adjusted for gestational age, maternal age, and parity was used when available to reduce confounding factors, this was not available in all cases.

## STAR★Methods

### Key resources table

**Table:** 

REAGENT or RESOURCE	SOURCE	IDENTIFIER
**Software and algorithms**		

GraphPad Prism 9	GraphPad Software	https://www.graphpad.com
BioRender	BioRender	https://www.biorender.com

### Resource availability

#### Lead contact

Further information and requests for resources should be directed to the lead contact, Professor Dino A. Giussani (dag26@cam.ac.uk).

#### Materials availability

This study did not generate new unique materials.

#### Data and code availability

• This paper analyses existing, publicly available data contained within the referenced papers.
• This paper does not report original code.
• Additional information required to reanalyse the data reported in this paper is available from the lead contact upon request.

### Method details

#### Paper selection

PubMed, Google Scholar and iDiscover were used to search for literature, with further examination of references to expand the identification process. Papers in which the data had been reported by a primary source elsewhere were excluded as well as papers omitting specific data values. Other limitations included lack of access and language barriers.

### Quantification and statistical analysis

Statistical analysis was performed using Prism 9 (GraphPad software). Statistical tests, parameters and outliers are reported in the figures and legends. For analysis, low birth weight was defined as birth weight <2500g and small for gestational age was defined as birth weight <10^th^ percentile after adjustment for gestational age. Where the altitude was given as a range, the middle of the range was used. If only an upper or lower boundary of altitude was given, then the data was discounted. If the study gave birthweight adjusted for gestational age, maternal age and parity then this was used. For birthweights divided by sex or ethnicity, the combined mean was used for the average birthweight at altitude.
